# Tobacco and e-cigarette shops awarded ‘essential business’ labels in France during COVID-19

**DOI:** 10.18332/tid/129269

**Published:** 2020-11-03

**Authors:** Alain Braillon

**Affiliations:** 1 Former senior consultant

**Keywords:** COVID-19, e-cigarettes, tobacco, public health

**Dear Editor,**

Egbe and Ngobese^1^ reported that South Africa and India banned the sale of tobacco products during lockdown periods against COVID-19. Vázquez and Redolar-Ripoll^2^ also previously called for strengthening population-based cessation support via: public campaigns, quitlines and nicotine replacement therapy availability. In contrast, in the US, Lang and Yakhkind^3^ used telehealth to scale up the tobacco cessation program in a military setting.

The inertia or lack of comprehensive measures against tobacco use during the COVID-19 pandemic is a concern as: a) the crisis not only has exacerbated risk factors for the initiation, worsening and relapse of addictive disorders but also seriously limited access to treatments; b) there has been accumulating robust evidence showing smoking worsens the progression and outcomes of viral or bacterial lung infections. Accordingly, the COVID-19 pandemic should have been a great lever for increasing motivation to quit. The state of affairs is similar for e-cigarettes, which may cause lung inflammation and increase susceptibility to pulmonary viral and bacterial infections^4-6^.

Only France had a comprehensive policy on tobacco during the pandemic, being among the first countries that implemented a severe lockdown, with the government issuing various levels of restrictions on non-essential activities and businesses. The Ministry of Health issued a legal decree^7^ explicitly allowing tobacco shops and e-cigarette shops to remain open, adding them to a detailed list of businesses previously allowed to operate on 14 March and ranking them just after ‘food retail trade on stalls and markets’. The latter were forbidden to operate on 23 March by the Prime Minister. On 23 April, the Ministry of Health suspended online sales of nicotine replacement therapy and restricted delivery by pharmacies to one month’s therapy^8^; while in parallel, the European ban on menthol cigarettes, which was to be enforced on 20 May 2020, was postponed to 31 July 2020.

In France, daily smoking prevalence was 24% in 2019, without significant changes from 2018. In light of the COVID-19 pandemic, could the government increase its revenue from tobacco taxes, which are equivalent to one-fourth of the revenue from income tax, in order to better face the COVID-19 induced economic crisis?
